# *In vivo* efficacy of sulphadoxine-pyrimethamine for the treatment of asymptomatic parasitaemia in pregnant women in Machinga District, Malawi

**DOI:** 10.1186/s12936-015-0710-7

**Published:** 2015-05-13

**Authors:** Julie Gutman, Dyson Mwandama, Ryan E Wiegand, Joseph Abdallah, Nnaemeka C Iriemenam, Ya Ping Shi, Don P Mathanga, Jacek Skarbinski

**Affiliations:** Malaria Branch, Division of Parasitic Diseases & Malaria, Centers for Disease Control and Prevention, Atlanta, GA USA; Malaria Alert Centre, University of Malawi College of Medicine, Blantyre, Malawi; Rwanda Zambia HIV Research Group, Emory University, Atlanta, GA USA; Department of Community Health, College of Medicine, Lilongwe, Malawi

**Keywords:** Malaria, Pregnancy, Intermittent-preventive treatment, Sulphadoxine-pyrimethamine, *in vivo*, Malawi

## Abstract

**Background:**

The effectiveness of sulphadoxine-pyrimethamine (SP) intermittent preventive treatment of malaria in pregnancy (IPTp) might be compromised by high prevalence of resistance-associated *Plasmodium falciparum* dihydrofolate reductase (*dhfr*) and dihydropteroate synthase (*dhps*) mutations. As a proxy for IPTp-SP effectiveness, the *in vivo* efficacy of SP to clear parasitaemia and prevent reinfection in asymptomatic parasitaemic pregnant women in an area with high SP resistance prevalence was assessed.

**Methods:**

Pregnant women 16–26 weeks’ gestation with asymptomatic parasitaemia presenting for antenatal care were given IPTp-SP and followed for 42 days. The primary outcome was polymerase chain reaction (PCR) uncorrected 42-day survival rate; the per cent of patients without recrudescence or reinfection by day 42. PCR was used to distinguish recrudescence from reinfection. DNA was sequenced to detect resistance-associated *dhfr* and *dhps* mutations.

**Results:**

Of 245 pregnant women included in the intention-to-treat analysis, 93.9% cleared their parasitaemia by day 7. The day 42 PCR-uncorrected survival rate was 58.1% (95% confidence interval (CI) 51.5-65.7) and day 42 PCR-corrected survival was 68.7% (CI 61.4-76.0). Recrudescence was more common among primi- than among multigravid women; recrudescence rate 33.3% (CI 25.1-42.4%) *versus* 21.4% (CI 15.0-29.0%) (log rank test p-value 0.006). The quintuple mutant was present in nearly all samples (95%), while 2% were sextuple mutants with an additional mutation at *dhps* A581G.

**Conclusions:**

SP efficacy for acute malaria treatment has been compromised by resistance, but SP retains partial activity among pregnant women with asymptomatic parasitaemia, and thus might be useful for IPTp. Nonetheless, research on non-SP IPTp regimens should continue.

**Trial registration:**

ClinicalTrials.gov NCT01120145.

## Background

Malaria in pregnancy is associated with severe maternal anaemia, placental malaria, low birth weight (LBW), maternal mortality, and infant morbidity and mortality, particularly among primigravid women [1]. Intermittent preventive treatment with sulphadoxine-pyrimethamine (IPTp-SP) reduces the adverse consequences of malaria among pregnant women, including third-trimester maternal anaemia, placental parasitaemia, and the prevalence of LBW infants [2-4]. When this study was conducted, the policy in Malawi was to give SP at the first antenatal care (ANC) visit after quickening (about 20 weeks) and again during the third trimester, with a total of two doses during pregnancy; this has since been updated in line with revised WHO recommendations to provide IPTp-SP at each scheduled ANC visit starting early second trimester with a minimum of three doses during pregnancy [5].

Resistance to SP is mediated by the sequential accumulation of single nucleotide polymorphisms (SNPs) that encode amino acid substitutions in the *Plasmodium falciparum* dihydropteroate synthase *(dhps)* and dihydrofolate reductase *(dhfr)* genes; the quintuple mutant haplotype (*dhfr* mutations N51I, C59R, S108N, and *dhps* mutations A437G and K540E), which is associated with failure of SP in *in vivo* studies in children, has been fixed in Malawi since 2005 [6,7]. Due to the high level of SP resistance, SP has not been recommended for treatment of symptomatic malaria in Malawi since 2007. Nonetheless, studies suggest that SP remains effective as IPTp even when it is no longer effective as a treatment in symptomatic children [2,8]. However, studies from both Tanzania and Malawi suggest IPTp-SP effectiveness could be compromised by the presence of additional mutations conferring increased resistance to SP [9-11]; in particular, the presence of an additional mutation at *dhps* A581G (the sextuple mutant) seems to significantly compromise the effectiveness of IPTp-SP [10,12,13].

As resistance to SP increases, it is important to monitor the efficacy of IPTp-SP to determine when this intervention no longer provides any benefit. However, there is no standardized methodology for monitoring. *In vivo* efficacy in children does not correlate well with the degree of protection afforded to pregnant women [2], because pregnant women have partial immunity, and thus other methods to monitor IPTp-SP effectiveness are needed. As a proxy for SP effectiveness, a 42-day *in vivo* efficacy study was conducted among asymptomatic pregnant women to assess the efficacy of SP for clearing existing parasitaemia and preventing new infection. As pregnancy specific anti-parasitic immunity increases with each pregnancy, efficacy was assessed separately among primigravid and multigravid women.

## Methods

### Study site

The study was conducted in the antenatal clinic of Machinga District Hospital, Malawi from March 2010 to January 2011. Residents are mainly of the Yao ethnic group, who earn their living through subsistence farming, fishing and small businesses. Machinga District Hospital is the primary hospital for a catchment area including 369,614 people, with approximately 5,712 deliveries per year. In 2011, there were 57,999 clinically diagnosed malaria cases, of which 29,626 (51%) were aged ≥ five years [12]. Malaria transmission in the district is stable throughout the year with a peak in the rainy season, from December to March. Parasite prevalence was 42.3% in children aged < five years in the Southern Region in the most recent malaria indicator survey [12]. The vast majority of malaria infections are caused by *Plasmodium falciparum*. Resistance to SP is widespread; the quintuple mutation is fixed, although at the time of this study the *dhfr* I164L mutation had not been detected and the *dhps* A581G mutation was detected at extremely low frequency (<1%) [6].

### Sample size

Parasitologic failure rate among pregnant women treated with SP was 11% in 2004, double the failure rate in 1996 (5%) [14]. Allowing for the possibility that failure rates had increased, a sample size of 300 women was determined to be sufficient to estimate the proportion of failures with a 95% confidence interval (CI) width of 0.10, assuming a 20% failure rate, and allowing for approximately 20% loss to follow-up [15].

### Enrolment and follow-up

Pregnant women of all parities presenting to Machinga District Hospital for ANC visits were eligible for inclusion if they: 1) were between 16 and 26 weeks’ gestation (based on last menstrual period (LMP) or quickening); 2) were eligible to take SP on the day of enrolment; 3) had malaria parasitaemia of any density; 4) had an axillary temperature below 37.5°C; 5) had a viable foetus documented by detection of a foetal heartbeat or foetal movement; 6) were HIV-negative; 7) had not received any anti-malarials or antibiotics with anti-malarial activity in the previous month; 8) had no history of SP hypersensitivity; and, 9) provided informed consent for participation. At enrolment, a finger-prick blood specimen was collected for malaria rapid diagnostic test (RDT) (Paracheck-Pf Device (Orchid Biomedical Systems, India)), malaria microscopy, haemoglobin measurement (HemoCue 201, HemoCue AB, Sweden), and dried blood spot (DBS) for polymerase chain reaction (PCR); three tablets of SP (a total of 1,500 mg sulphadoxine and 75 mg of pyrimethamine) was administered by directly observed therapy. Quality-assured SP was procured from Durbin Plc, Middlesex, UK.

Following enrolment, patients were assessed weekly until day 42 or until they developed parasitaemia. At each visit, women were assessed for fever (axillary temperature ≥37.5°C) or histoy of fever and medication use, and a finger-prick blood specimen was collected for thick smear, haemoglobin measurement, and DBS for PCR to distinguish reinfection from recrudescence. Thick blood smears were prepared with Field’s stain and the number of parasites per 200 white blood cells (WBC) counted. Blood smears were considered negative if no parasites were found after counting 1000 WBC. Blood smears were read by two independent microscopists with a third read in the case of discordant results (discrepancy between positivity/negativity or greater than 50% difference in parasite counts). Parasite density was calculated as the average of the closest two readings, assuming an average WBC count of 8,000/mm^3^. Participants were instructed to return to the clinic immediately if they felt sick. Women with positive blood smears at any time on or after day 4 were treated with artemether-lumefantrine per national guidelines.

### Molecular analyses

Resistance to SP was measured by direct sequencing of DNA extracted from DBSs collected from the day of enrolment. The DNA samples were sequenced at *dhfr* codons 51, 59, 108, 164 and *dhps* codons 436, 437, 540, 581, 613 using a standard protocol [16]. The quintuple mutant was defined as an isolate containing *dhfr* mutations N51I, C59R and S108N, and *dhps* mutations A437G and K540E [17]. The presence of an additional mutation at *dhps* A581G defined the sextuple mutant.

In order to differentiate recrudescence from reinfection, DNA samples extracted from paired DBS samples from enrolment (day 0) and day of failure were assessed using WHO-recommended PCR procedures for testing polymorphisms at the genes encoding merozoite surface protein 1 (*msp*-1) and *msp*-2 [18].

### Statistical analysis

As IPTp-SP is designed to both treat prevalent parasitaemia as well as prevent incident parasitaemia, the main outcome of interest was clearance of parasites without recrudescence or reinfection in the 42 days following SP administration (survival), which differs from the standard WHO *in vivo,* in which the main outcome of interest is clearance of parasites without recrudescence (i.e. treat prevalent infection only) [15]. Data analysis was done in SAS version 9.3 (Cary, NC, USA) and R version 3.0.1 (Vienna, Austria). Survival curves were calculated using the Kaplan-Meier method; in absence of failure, women were censored on the last day for which data were available [19]. The log-rank test was used to assess for significant differences between curves. Multiple imputation (with 10 imputed datasets) was used to assess the sensitivity of results when incorporating cases where PCR testing for recrudescence *versus* reinfection was unsuccessful [20]. Cox proportional hazards regression was used to test for predictors of failure [21]. The final, multivariable model was chosen to reflect predictors which improve the fit of the model based on Schwarz’s Bayesian Information Criterion [22]. For all analyses, a p-value of <0.05 considered significant. Plots were generated with the ggplot2 package [23].

### Ethics

The study was approved by the ethical review boards of the University of Malawi College of Medicine (Blantyre, Malawi) and the Centers for Disease Control and Prevention (Atlanta, GA, USA). Written informed consent was obtained from all participating women prior to enrolment. The study was registered at ClinicalTrials.gov (NCT01120145).

## Results

**Figure 1:**
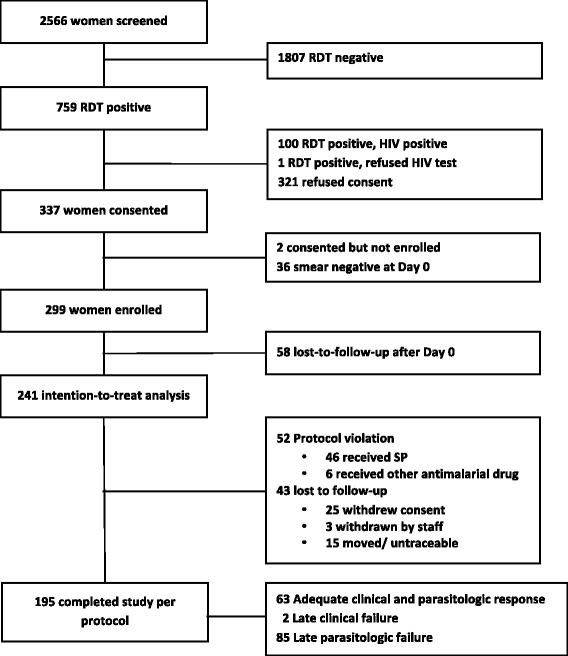
Enrolment flow diagram for sulphadoxine-pyrimethamine (SP) modified *in vivo* efficacy study in pregnant women*. *Study included pregnant women with asymptomatic malaria parasitaemia attending antenatal care for their first dose of intermittent preventive treatment in pregnancy with SP (IPTp-SP), Machinga District Hospital, Malawi 2009–2010. RDT = rapid diagnostic test.

A total of 2,566 women were screened; *P. falciparum* antigenaemia was detected by RDT in 759 (29.6%). Of the 301 women who met all inclusion criteria and consented to enrolment, 299 were enrolled; 58 women were lost prior to the first follow-up, and were excluded from the analysis. A total of 245 pregnant women were included in the analyses (Figure 1). Of these, 114 (46.5%) were primigravid (G1) and 131 (53.5%) were multigravid (G2+) (Table 1). The mean age was 21.7 years (range 14–42) overall, 18.8 years (range 14–38) among primigravid women and 24.3 years (range 16–42) among multigravid women; 54.3% were less than or equal to 20 years old. In all, 32.2% reported having used a bed net on the night prior to enrolment; 84.8% of these were insecticide-treated nets (ITNs). Bed net use was higher among multigravid than among primigravid women (41.2 *versus* 21.9%, p = 0.001). The mean gestational age at enrolment was 21 weeks (IQR 18–24); multigravid women were enrolled on average 1.5 weeks later in gestation than primigravid women (p = 0.002).

**Table 1:** **Baseline characteristics of asymptomatic parasitaemic pregnant women enrolled in a modified** ***in vivo*** **efficacy study of sulphadoxine-pyrimethamine (SP), stratified by gravidity**

	**Total (N = 245)**	**Primigravid (N = 114)**	**Multigravid (N = 131)**	**p-value**
**Age, mean (SD)**	21.7 (5.0)	18.8 (2.9)	24.3 (5.0)	<0.0001
**Age** ≤20 **years**	54.3%	87.7%	25.2%	<0.0001
**Bed net use**				
Used a bed net last night	32.2%	21.9%	41.2%	0.001
Used an insecticide-treated bed net last night	27.3%	14.9%	38.2%	<0.0001
**Maternal weight (kg), mean (SD)**	55.8 (7.1)	55.4 (6.9)	56.1 (7.3)	0.40
**Education (years), mean (SD)**	6.2 (3.0)	6.9 (2.9)	5.6 (3.0)	<0.001
**Perceived below average wealth status**	78.0%	81.6%	74.6%	0.19
**Gestational age at enrolment (weeks), mean (SD)**	21.0 (3.9)	20.2 (3.6)	21.8 (4.0)	0.002
**Haemoglobin at enrolment, mean (SD)**	9.8 (1.3)	9.2 (1.1)	10.4 (1.3)	<0.0001
**Per cent of women with anaemia (Hb <11 g/dL) at enrolment**	81.2%	96.5%	67.9%	<0.0001
**Per cent of women with gametocytaemia**	0.4%	0.00%	0.8%	-
**Parasite density (parasites/ml), geometric mean (range)**	273 (32–12,835)	372 (48–12,835)	209 (32–8,955)	<0.0001

SD = standard deviation, Hb = haemoglobin.

### Recrudescence and reinfection

During the course of the study, 63 women (25.7%) remained aparasitaemic through day 42 while 87 women developed parasitaemia (35.5%) of whom 51 (58%) had recrudescent infection, 17 (20%) were reinfected, and in 19 (22%) PCR was unavailable (13 samples could not be amplified and six were unavailable). Reinfection occurred at a median of 15 days (interquartile range 14 – 28 days). A total of 95 women were censored prior to reaching a valid study end point; 43 women were lost to follow-up, and another 52 women were withdrawn due to protocol violation (46 were given IPTp-SP at day 35 and six received anti-malarial treatment without evidence of malaria or another antibiotic with anti-malarial activity).

**Figure 2:**
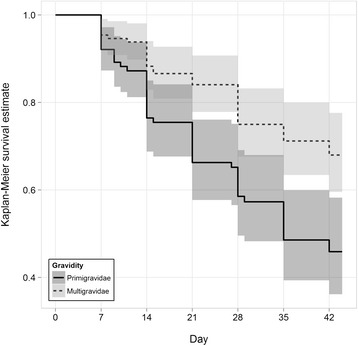
Kaplan-Meier survival curve estimates for asymptomatic parasitaemic women following treatment with sulphadoxine-pyrimethamine at day 0 with 95% confidence intervals, by gravidity.

Overall, 93.9% of women cleared their infection by day 7; the PCR-uncorrected 28-day survival rate was 67.5% (Table 2). The PCR-uncorrected 42-day survival rate was 58.1%; the survival rate was 45.8% in primigravid women and 68.0% in multigravid women. Over 42 days, there was a significant association between survival and gravidity (log-rank Chi-sq = 11.5, df = 1, p = 0.0007) (Figure 2). PCR-corrected survival, including multiple imputations for missing PCR results, was 76.4% (CI 70.2-82.6) at day 28 and 68.7% (CI 61.4-76.0) at day 42. Recrudescence was more common among primi- than among multigravid women, with a recrudescence rate of 44.0% (CI 31.6-56.3%) and 23.6% (CI 14.9-32.3%), respectively (log-rank Chi-sq = 9.3, df = 1, p = 0.002) by day 42.

**Table 2:** **Polymerase chain reaction survival rates by week among asymptomatic parasitaemic women treated with sulphadoxine-pyrimethamine at day 0, stratified by gravidity**

**Uncorrected**			
**Day of follow-up**	**Primigravid survival rate (95% CI)**	**Multigravid survival rate (95% CI)**	**Overall survival rate (95% CI)**
**7**	92.1% (87.3-97.2)	95.4% (91.9-99.1)	93.9% (90.9-96.9)
**14**	76.5% (68.8-85.0)	88.3% (82.9-94.0)	82.9% (78.2-87.9)
**28**	58.5% (49.6-69.1)	75.0% (67.6-83.2)	67.5% (61.6-74.0)
**35**	48.6% (39.3-60.0)	71.2% (63.4-79.9)	61.1% (54.8-68.1)
**42**	45.8% (36.1-58.2)	68.0% (59.5-77.6)	58.1% (51.5-65.7)
**Corrected**			
**Day of follow-up**	**Primigravid survival rate (95% CI)**	**Multigravid survival rate (95% CI)**	**Overall survival rate (95% CI)**
**7**	95.9% (91.0, 100.0)	97.1% (93.9, 100.0)	96.5% (93.6, 99.4)
**14**	84.2% (76.5, 91.9)	91.4% (86.2, 96.7)	88.2% (83.6, 92.8)
**28**	68.3% (58.3, 78.3)	82.8% (75.5, 90.1)	76.4% (70.2, 82.6)
**35**	59.3% (48.1, 70.5)	78.6% (70.6, 86.6)	70.2% (63.5, 77.0)
**42**	56.0% (43.7, 68.3)	76.4% (67.7, 85.0)	68.7% (61.4, 76.0)

CI: confidence interval.

Both higher gravidity and bed net use were shown to be protective against developing recrudescence or reinfection in univariate modelling, however, in the multivariate model, only gravidity remained significant (Table 3). When examining the risk of recrudescence only, as expected, bed net use was no longer a significant factor, and only gravidity remained significant in both univariate and multivariate analyses.

**Table 3:** **Factors associated with treatment failure among asymptomatic parasitaemic women treated with sulphadoxine-pyrimethamine at day 0**

	**Univariable**				**Multivariable**			
		**95% CI**				**95% CI**		
	**HR**	**Lower**	**Upper**	**p-value**	**HR**	**Lower**	**Upper**	**p-value**
**Treatment failure, uncorrected by PCR**								
**ITN use last night (reference: no ITN use last night)**	0.54	0.32	0.92	0.02	0.67	0.38	1.16	0.16
**Age: comparing >20 years to ≤20 years**	0.68	0.44	1.06	0.09				
**Schooling years** (continuous)	1.01	0.94	1.09	0.72				
**Log parasite density** (continuous)	0.96	0.79	1.17	0.70				
**Gravidity: comparing multigravid to primigravid**	0.48	0.31	0.74	<0.001	0.53	0.34	0.83	0.006
**Treatment failure, corrected by PCR**								
**ITN use last night (reference: no ITN use last night)**	0.54	0.27	1.09	0.09	0.73	0.35	1.52	0.40
**Age: comparing >20 years to ≤20 years**	0.62	0.35	1.10	0.10				
**Schooling years** (continuous)	1.05	0.95	1.15	0.35				
**Log parasite density** (continuous)	0.97	0.75	1.25	0.79				
**Gravidity: comparing multigravid to primigravid**	0.41	0.23	0.73	0.002	0.45	0.25	0.82	0.009

HR = hazard ratio, CI = confidence interval, ITN = insecticide-treated bed net, PCR = polymerase chain reaction.

### Haematologic response

**Figure 3:**
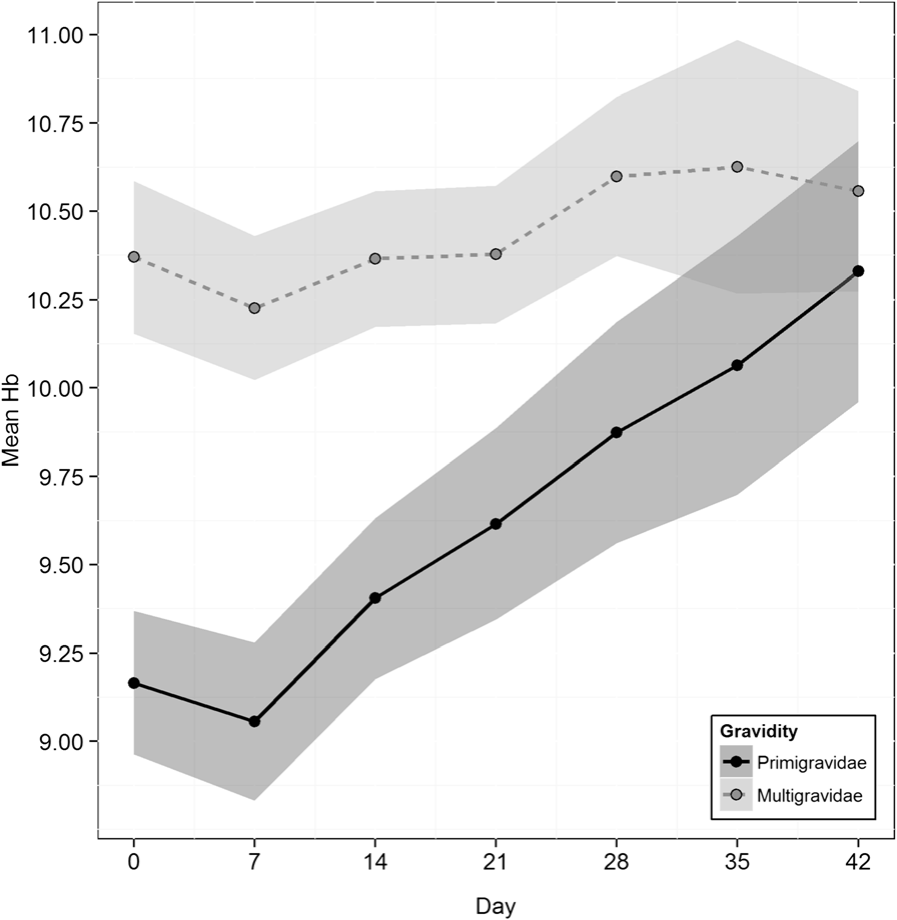
Mean haemoglobin levels with 95% confidence intervals broken down by gravidity.

Mean haemoglobin increased by 0.3 mg/dL (95% CI 0.1-0.4, p = 0.0001) from day 0 until the last day of follow-up. The change was greater among primigravid women (0.4 mg/dL (95% CI 0.2-0.7) than among multigravid women (0.1 mg/dL (95% CI −0.05- 0.3), p = 0.02). A significant interaction was found between day of follow-up and gravid class (F(6,243) = 3.86, p = 0.0011) indicating the change in haemoglobin levels during the study was not the same in each group. Figure 3 shows the raw means with 95% confidence intervals across the two groups.

### Molecular markers of SP resistance

Of the 245 day 0 samples, a variable number could be amplified at each codon. Where samples were amplified, there was a very high frequency of mutation (≥97%) at each of the following codons: *dhfr*-51, *dhfr*-59, *dhfr*-108, *dhps*-437, and *dhps*-540 (Table 4). Of 182 samples that were successfully genotyped at all five codons, 95% carried the quintuple mutant. Four samples (2%) (one from a primigravid woman and three from multigravid women), all of which carried the quintuple mutation, were mutated at *dhps-*581 indicating the presence of sextuple mutants. One of these patients had adequate clinical and parasitologic response (ACPR), two had a treatment failure (one on day 7 and the other on day 8), and one was censored on day 35 due to a protocol violation. The presence of the sextuple mutant was detected in three additional samples from the day of failure (one from a primigravid woman and two from multigravid women). Unfortunately, the day-of-failure samples from the two patients who had sextuple mutants detected at baseline and subsequently failed therapy could not be amplified at codon *dhps*-581.

**Table 4:** **Prevalence of mutations** ***Plasmodium falciparum*** **dihydrofolate reductase (** ***dhfr*** **) and dihydropteroate synthase (** ***dhps*** **) mutations associated with resistance to sulphadoxine-pyrimethamine detected at day 0**

**Mutation**	**Mutant (n)/Total (N)**	**Per cent mutant**
***dhfr*** **N51I**	209/212	99%
***dhfr*** **C59R**	210/214	98%
***dhfr*** **S108N**	211/214	99%
***dhfr*** **I164L**	2/211	1%
***dhps*** **A437G**	219/225	97%
***dhps*** **K540E**	201/204	99%
***dhps*** **A581G**	4/204	2%
***dhps*** **A613T/S**	2/180	1%
Double*	187/193	97%
Triple*	196/202	97%
Quintuple*	172/182	95%

*Double mutant = *Pfdhps* mutations A437G and G540E; Triple mutant = *Pfdhfr* mutations N51I, C59R and S108N; Quintuple mutant = *Pfdhfr* mutations N51I, C59R, S108N and *Pfdhps* mutations A437G and K540E.

## Discussion

Overall, 58.1% of pregnant women who presented with asymptomatic parasitaemia and received SP on day 0 remained aparasitaemic for 42 days, in an area with a high prevalence of SP resistance (95% carried the quintuple mutant, 2% carried the sextuple mutant at baseline). Parasite-free survival rates were higher among multigravid (68%) than among primigravid (46%) women. Although 28-day survival was 67.5%, well below the 90% WHO threshold for changing the recommendation for first-line treatment of uncomplicated malaria [24], this study suggests that SP retains some efficacy among pregnant women, and that monthly dosing of IPTp-SP, as recommended by WHO [25], likely continues to provide benefit. This is supported by a recent study conducted at the same site assessing the effect of IPTp-SP on birth outcomes, which found a dose-dependent protective effect of IPTp-SP among primigravid women on a composite birth outcome of LBW, preterm delivery, and small for gestational age (SGA) [26]. Similarly, a recent meta-analysis found a greater benefit of IPTp-SP when three or more doses were given during pregnancy compared to two, irrespective of the prevalence of the quintuple mutation [8].

Although the presence of the quintuple mutant predicts failure of SP treatment among children aged < five years with acute malaria [17], IPTp-SP remains effective even where the quintuple mutant is highly prevalent [8]. In this study, due to the extremely high prevalence of the quintuple mutant, it was not possible to assess whether this haplotype was associated with a higher rate of failure; despite the high prevalence of the quintuple mutant, IPTp-SP continues to improve birth outcomes in this area [26]. In Tororo, Uganda, where >90% of parasites carry the quintuple mutant, Arinatwe *et al*. also found a significant reduction in a composite adverse outcome measure composed of placental malaria, LBW, maternal parasitaemia, or maternal anaemia [27]. A study assessing SP *in vivo* efficacy among asymptomatic, parasitaemic pregnant women in Zambia, where the quintuple mutant was present in 61% of samples at baseline, found no association of the quintuple mutant with treatment failure [28]. In that study setting, IPTp-SP was associated with a dose-dependent reduction in LBW, with a greater impact among primi- and secundigravid compared to multigravid women [29].

Although IPTp-SP remains effective even in the presence of the quintuple mutant, several studies suggest that the presence of the sextuple mutant is associated with decreased effectiveness of IPTp-SP, as evidenced by either increased parasite densities, increased placental inflammation, or decreased birth weight among children born to women infected with parasites carrying the sextuple mutant [10,12,13]. In an area with extremely high levels of resistance in Muheza, Tanzania, where the quintuple mutant is fixed and the mean fraction of resistant alleles at *dhps 581* was 0.31, IPTp-SP was not associated with a statistically significant benefit at the population level [11]. The lack of efficacy may reflect the effect of the high prevalence of the sextuple mutant or alternatively may have been due to insufficient power in the study, as the point estimates for the effect on LBW favour IPTp. As the prevelance of SP resistance continues to increase, additional studies are needed to assess the true effect of the sextuple mutant on IPTp-SP efficacy, and the prevalence at which a population level effect is seen.

The protective effect of IPTp-SP is thought to be a result of both the ability to clear existing infections as well as to prevent new infections. Some have argued that the prophylactic effect is of primary importance [30]. However, a recent study from Malawi found that IPTp-SP was more effective in clearing existing sub-microscopic infections than preventing new sub-microscopic infections [31]. Moreover, intermittent screening with a RDT and treatment only of parasitaemic women has been shown to have similar benefits to IPTp-SP in preventing LBW and severe maternal anaemia, even when using a treatment with a short-acting drug such as artesunate-amodiaquine or artemether-lumefantrine [32]. (Tagbor et al., A non-inferiority, individually randomized trial of intermittent screening and treatment versus intermittent preventive treatment in the control of malaria in pregnancy, submitted). This suggests that the treatment effect might be more important than the prophylactic effect. In this study, only 6.1% of women had persistent infection on day 7, indicating that the vast majority of infections were suppressed, despite the high prevalence of parasite resistance to SP. SP was effective at clearing infections likely due to the fact that the infections were of low density; however immunity also played a role given the differential rate of cure between primi- and multigravid women.

Across the reported *in vivo* efficacy studies among pregnant women, there appears to be a correlation between failure rate and the prevalence of SP resistance mutations (Desai M, et al.: Impact of sulfadoxine-pyrimethamine resistance on effectiveness of intermittent preventive therapy for malaria in pregnancy at clearing infections and preventing low birth weight: A prospective, multi-country observational study across Africa, submitted). In a recent study in Mali and Burkina Faso, where the triple *dhfr* mutant was detected in <50% and the *dhps* K540E mutation was detected in less than 1% of samples, the overall PCR uncorrected failure rate was 4.9% [33]. In Benin, where the *dhfr*/*dhps* quadruple mutant was detected in >80% of parasite isolates, but *dhps* K540E was not detected, 11% of women with positive smear at the time of IPTp-SP administration had parasites detected by microscopy on follow-up within one month [34]. In Zambia, where the *dhfr*/*dhps* quintuple mutant was detected in parasites in 63% of women, failure rates were 26% by 35 days, [28] while in this site in Malawi, where parasites from 95% of women carried the quintuple mutant, failure rates were 32.5% at 28 days and 38.9% by day 35. While failure rates in these modified *in vivo* efficacy studies appear to correlate quite well with the prevalence of SP resistance mutations, there is poor correlation between SP resistance and IPTp-SP efficacy; the effect of IPTp-SP on infant birth weight or maternal haemoglobin remains remarkably constant over the spectrum of resistance, up to fixation of the quintuple mutant (Desai M, et al.: Impact of sulfadoxine-pyrimethamine resistance on effectiveness of intermittent preventive therapy for malaria in pregnancy at clearing infections and preventing low birth weight: A prospective, multi-country observational study across Africa, submitted). There are some data to suggest that the additional mutation at *dhps* A580G renders IPTp-SP ineffective, however, additional studies are needed to confirm this relationship. Given the expense and difficulties associated with conducting *in vivo* efficacy studies, and the correlation of failure rates with SP resistance mutations, consideration should be given to monitoring SP resistance markers instead of conducting *in vivo* efficacy studies. However, until a clearer relationship exists between the prevalence of molecular markers of SP resistance and the threshold at which IPTp-SP is no longer effective, IPTp-SP should continue to be delivered, ideally at each scheduled ANC visit, with close monitoring in areas where the sextuple mutant is emerging.

### Limitations

Although this study suggests a beneficial effect of SP, the lack of a placebo arm impedes the ability to assess the true effect of SP treatment. It is possible that some women, particularly the multigravid ones, would have been able to clear the infections from their peripheral blood even without being treated with SP. However, until there is more evidence that IPTp-SP does not provide any benefit, it is not ethical to conduct such a study. Furthermore, as these women were not followed until delivery, it is not known whether the infections were cleared from the placenta, which remains a relatively protected site for malaria.

## Conclusion

Despite fixation of the quintuple mutant in this population, SP retained some benefit when administered to asymptomatic parasitaemic pregnant women. The difference in parasite clearance in multigravid *versus* primigravid women highlights the importance of pre-existing immunity in clearing parasites. Although the prevalence of SP resistance has compromised the efficacy of SP as a treatment for acute malaria, it retains partial activity among pregnant women with asymptomatic parasitaemia, and is still useful when given as IPTp. Nonetheless, it remains important to continue to search for alternative regimens to prevent malaria in pregnancy.

## Abbreviations

ANC: Antenatal care
CI: Confidence interval
dhfr: Dihydrofolate reductase
dhps: Dihydropteroate synthase
IPTp: Intermittent preventive treatment of malaria in pregnancy
IQR: Inter-quartile range
ITN: Insecticide-treated nets
LBW: Low birth weight
msp: Merozoite surface protein
PCR: Polymerase chain reaction
SP: Sulphadoxine-pyrimethamine
WHO: World Health Organization
